# Single neuron activity and c‐Fos expression in the rat striatum following electrical stimulation of the peripheral vestibular system

**DOI:** 10.14814/phy2.13791

**Published:** 2018-07-12

**Authors:** Lucy Stiles, John N. Reynolds, Ruth Napper, Yiwen Zheng, Paul F. Smith

**Affiliations:** ^1^ Department of Pharmacology and Toxicology University of Otago Dunedin New Zealand; ^2^ Department of Anatomy School of Biomedical Sciences University of Otago Dunedin New Zealand; ^3^ Brain Health Research Centre University of Otago Dunedin New Zealand; ^4^ Brain Research New Zealand Centre of Research Excellence University of Auckland Auckland New Zealand; ^5^ Eisdell Moore Centre for Hearing and Balance Research University of Auckland Auckland New Zealand

**Keywords:** c‐Fos, single neurons, striatum, vestibular

## Abstract

Connections between the vestibular system and the basal ganglia have been postulated since the early 20th century. However, the results of electrophysiological studies investigating neuronal responses to electrical stimulation of the vestibular system have been inconsistent. The aim of this study was to investigate the effects of electrical stimulation of the vestibular labyrinth on single neuron activity and c‐Fos expression in the rat striatum. We used electrical stimulation of the vestibular labyrinth (various intensities delivered to the round window) to examine the electrophysiological response of striatal neurons and c‐Fos expression. From 507 single neurons recorded (*n *=* *20 rats), no vestibular‐responsive neuron was found at 1× and 2× the nystagmus threshold; however, 6 neurons were found at 3× the threshold. These neurons were found bilaterally, with a response latency of ~50 msec from the end of the stimulus. For the c‐Fos study, the number of neurons expressing c‐Fos was quantified using stereological methods. Stimulation at 2× the threshold for nystagmus (*n *=* *5 rats) resulted in a significant decrease in the number of neurons expressing c‐Fos in the bilateral striatum compared to both the sham control group (*n *=* *5) and the lower stimulus intensity group (*n *=* *5) (*P *≤* *0.0001 for both). The results of this study demonstrate that: (1) some single striatal neurons respond to electrical vestibular stimulation, however, these responses are circumscribed and infrequent; (2) electrical stimulation of the vestibular labyrinth results in a decrease in the number of striatal neurons expressing c‐Fos, in a current‐dependent manner.

## Introduction

Since the first half of the 20th century, it has been hypothesized that the vestibular system might transmit sensory information to the striatum (e.g., Muskens 1914, 1922). It seemed self‐evident that a sensory system that detects angular and linear acceleration of the head during self‐motion (see Cullen 2012 for a review), might provide useful sensory data to a CNS structure concerned with the control of voluntary movement (see Stiles and Smith 2015 for a review). Nonetheless, convincing evidence of such a connection has been slow to emerge.

It was suggested that vestibular information might be transmitted to the striatum via the motor cortex (e.g., Garcia‐Rill 1986) or the hippocampus (e.g., Kelley and Domesick 1982) and more recently, it has been suggested that there may be more direct pathways via the parafascicular nucleus (PFN) of the thalamus (Lai et al. 2000; see Stiles and Smith 2015 for a review; Kim et al. 2017). Potegal et al. (1971) sought to confirm Muskens' hypothesis (e.g., Muskens 1914, 1922) that vestibular information was transmitted to the caudate nucleus of the striatum via subcortical pathways, by lesioning the “vestibular cortical projection area” and recording from the caudate nucleus during electrical stimulation of the vestibular nerve. These lesions resulted in no change in the evoked field potentials, suggesting that there may indeed be subcortical pathways. Later studies demonstrated evoked field potentials in both the caudate nucleus and putamen of the striatum in response to electrical stimulation of the vestibular nerve in squirrel monkeys (Liedgren and Schwarz 1976) and the lateral and medial vestibular nuclei in cats (Spiegel et al. 1965). However, compared to field potential studies, very few single neuron studies have been conducted. It has been reported that electrical stimulation of the vestibular labyrinth caused an increase in the firing rate of single neurons in the putamen and the globus pallidus in cats (Segundo and Machne 1956). However, electrical stimulation of the contralateral vestibular nucleus in awake rhesus monkeys resulted in no change in the firing of single striatal neurons in the caudate nucleus, except when stimulation trains were used and the current intensity was high enough to produce movement of the limbs (Matsunami and Cohen 1975). More recently, Rancz et al. (2015) have investigated electrical stimulation of the superior vestibular nerve in rats and found that field potentials and multi‐unit activity could be evoked in the striatum. In the same study they confirmed this result using fMRI. Neurons in the striatum have been demonstrated to respond to movements in a way that is in phase with head velocity, possibly reflecting a vestibular influence (Barter et al. 2014; Kim et al. 2014). PET and fMRI studies in humans have demonstrated increases in activity in the putamen and the caudate nucleus, following either cold caloric vestibular stimulation or galvanic vestibular stimulation (GVS) (Bottini et al. 1994; Vitte et al. 1996; Emri et al. 2003; Della‐Justina et al. 2014). Most recently, it has been reported that people with persistent postural perceptual dizziness (PPPD) exhibit a decrease in gray matter volume in the caudate nucleus (Wurthmann et al. 2017).

**Figure 1:**
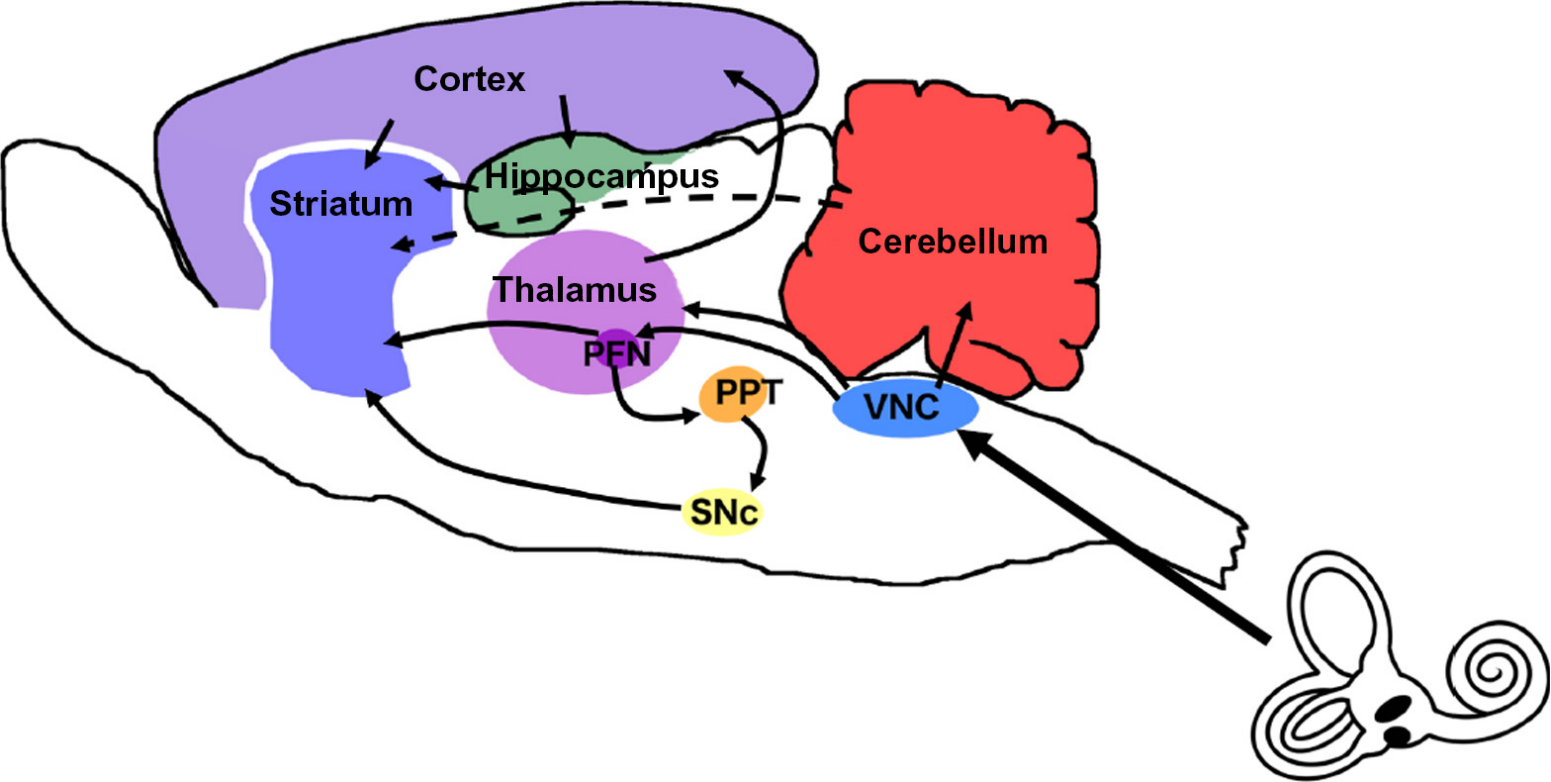
Possible neuronal pathways connecting the vestibular nucleus complex to the striatum. PFN, parafascicular nucleus; PPT, pedunculopontine tegmental nucleus; SNc, Substantia nigra pars compacta; VNC, vestibular nucleus complex.

Using neuronal tracers, Lai et al. (2000) demonstrated afferent projections from the medial vestibular nucleus to the PFN, and that these PFN neurons projected to the dorsolateral putamen. On the basis of these results they suggested that there may be a disynaptic pathway from the vestibular nucleus to the striatum via the PFN (see Fig. 1 for a summary). Kim et al. (2017) electrically stimulated the horizontal semi‐circular canal vestibular nerve in rats and found that they could evoke polysynaptic field potentials in the PFN, predominantly contralateral to the stimulus.

The possible contribution of vestibular input to the striatum has stimulated considerable interest in relation to Parkinson's Disease and hyperactivity syndromes (e.g., Antoine et al. 2013), and there have been attempts to use galvanic vestibular stimulation to treat the symptoms of Parkinson's Disease, in which balance deficits are particularly problematic (Yamamoto et al. 2005; Pan et al. 2008; Samoudi et al. 2012; Iwasaki et al. 2014). Therefore, a better understanding of the possible influence of the vestibular system on the striatum is needed. The available electrophysiological evidence is contradictory, especially in terms of the few single neuron recording studies. Therefore, the objectives of this study were to determine: (1) whether single neurons in the striatum would respond to electrical stimulation of the peripheral vestibular system in urethane‐anesthetized rats; (2) whether electrical stimulation of the peripheral vestibular system in rat resulted in a change in the number of cells expressing c‐Fos, using the immediate early gene protein c‐Fos as a marker of cellular activation in the striatum (Sheng and Greenberg 1990).

## Methods

### Animals

For the electrophysiological study, data were collected from male Wistar rats (*n *=* *20) weighing between 250 and 400 g. For the c‐Fos study, male Wistar rats weighing between 300 and 400 g were randomly allocated (*n *=* *5 per group) to the sham (Group 1) or stimulation groups (Groups 2 and 3). Prior to surgery, animals were maintained on a 12 h light‐dark cycle with free access to food and water. All procedures were approved by the University of Otago Animal Ethics Committee.

#### Electrical stimulation and surgery

**Figure 2:**
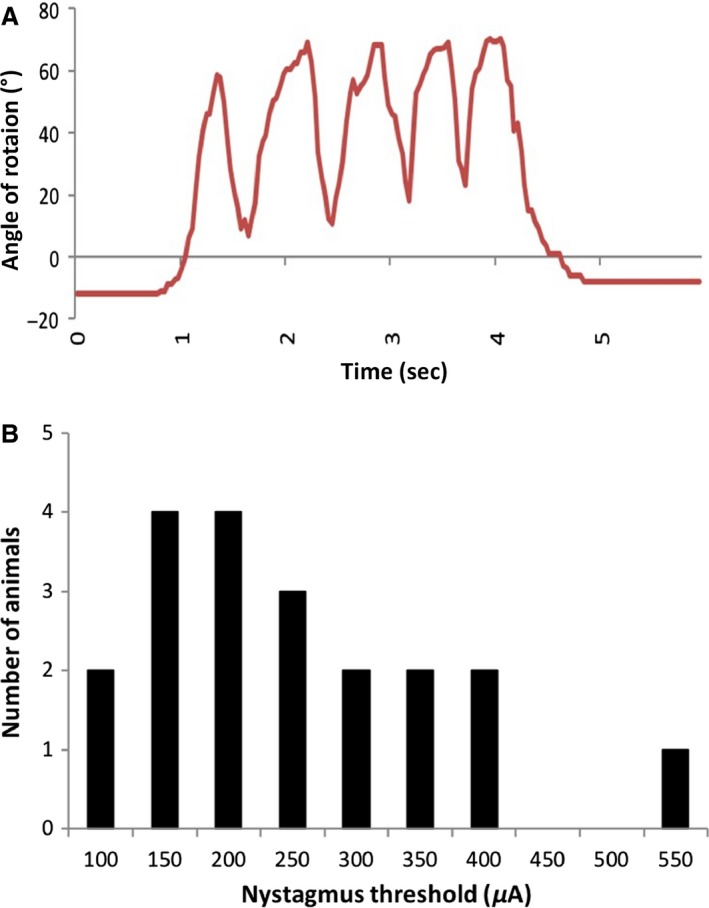
(A) Example of an eye movement trace of the ipsilateral eye in response to vestibular stimulation in a single rat. The animal received five 1 s stimulations (100 Hz, 400 *μ*A) with 0.5 s in between. Eye movement was measured as the change in the angle of rotation from the “zero” around the pupil of the eye. (B) Threshold for nystagmus production in urethane‐anesthetized rats undergoing electrical vestibular stimulation at 100 Hz. Data are shown as number of animals with thresholds as specific amplitudes (*μ*A) (*n *=* *22).

The procedure used to implant vestibular stimulating electrodes into the round window was identical for both parts of the study. In order to determine the optimal stimulation frequency, a range of frequencies was tested (0.1, 1, 10, 100 Hz) which covered much of the range used in the literature (e.g., Potegal et al. 1971; Liedgren and Schwarz 1976; Courjon et al. 1987; Anker et al. 2003). Each frequency was tested at multiple intensities from 50 *μ*A up to 2 mA or until nystagmus was visible. Only stimulation trains were used because single pulses have been shown not to activate the vestibular pathways (Potegal et al. 1971; Courjon et al. 1987; Anker et al. 2003). Contrary to previous studies, nystagmus was seen only at 100 Hz and not at any other frequencies tested; therefore, this frequency was used. At 100 Hz the threshold for the visualization of nystagmus was between 200 and 400 *μ*A for most animals (see Fig. 2). Electrical stimulation was controlled using Spike‐2 software (Cambridge Electronic Design, Cambridge, England) and the stimulation current was produced using an analogue stimulation isolator (Model 2200: A‐M systems). Although the aim of the studies was to use the lowest current amplitude possible, in order to be sure that the vestibular system was activated, we needed to establish the threshold for the induction of vestibular nystagmus. If electrophysiological responses were not obtained at this threshold, then the current amplitude was increased (i.e., up to 3× the threshold) in order to determine whether responses could be obtained at all. This procedure, of course, increased the risk of nonselective stimulation of other sensory systems such as the auditory system; however, electrical stimulation of the round window has been reported not to activate single neurons in the auditory cortex or to induce auditory sensations in humans (e.g., Korhuber and DaFonseca 1964; Schwartzkroin 1973) and in this, case bipolar electrodes, positioned on the round window, were used.

The animals were anesthetized with i.p urethane (1.5–2.0 g/kg). Xylocaine (with 1: 10,000 adrenaline, 0.5 mL, s.c.) was injected around the wound margins before any incisions were made. Areflexia was assessed by an absent response to a toe‐pinch. During the surgery the animal's body temperature was monitored using a rectal probe (Harvard Apparatus) and maintained at 37°C. Once anesthetized, the surgical site was shaved and the animal placed into a custom‐made nose bar. The surgical procedure was performed under an otolaryngological microscope (OPMI Pico, Zeiss, Hamburg, Germany). The tympanic bulla was exposed using a retro‐auricular surgical approach and a dental drill used to open the bulla to expose the round window. A stainless‐steel bipolar electrode (MS303/1‐B/SPC, Plastics One Inc.), insulated except at the tip, was placed into the round window as the stimulating electrode. The electrode was secured in place using dental cement, once the location of the electrode placement was confirmed via visualization of vestibular nystagmus in response to stimulation. This was done using a Dino‐Lite microscopic video camera focused on the rat's eye (Zheng et al. 2014), images from which were displayed on a PC. For each rat the threshold for nystagmus was defined as the lowest current at which eye movement was visible. These thresholds were used to establish the stimulus currents used in the studies. Analysis of the video files of the animals' eye movements was performed using eye tracking software (AET Tracker, STARNAV, France; see Fig. 2).

### Single neuron recording study

#### Electrophysiological recordings

The animal was transferred to a stereotaxic frame for single‐unit recordings. An incision was made down the midline of the scalp to expose the skull. A Ag/AgCl pellet electrode (Warner Instruments, Hamden, CT), used as the reference electrode, was inserted under the skin in the back of the animal's neck. Craniotomies were performed to expose the area (~2 mm diameter) of the brain containing the striatum either ipsilateral or contralateral to the stimulating electrode (0.0 to 1.5 mm AP and + or − 2 to 3 mm ML) (Paxinos & Watson, 2007). Burst trains of 0.5 s at 100 Hz every 4 secs at either 1×, 2× or 3× the threshold for nystagmus, were used to search for vestibular‐responsive neurons. Current amplitudes 3× the nystagmus threshold were used because no responses were found at the threshold current and Rancz et al. found that higher currents were necessary to evoke field potential and multi‐unit responses in the striatum (personal communication; Rancz et al. 2015).

Single‐unit responses in the striatum were recorded through glass micropipettes made from borosilicate glass capillaries (Harvard Apparatus, Harvard, MA). Electrodes were pulled and filled immediately before recording. The micropipettes filled with 1 M NaCl, and a silver chloride wire (12–18 MΩ), were connected to a headstage (NL100RK, Digitimer, Hertfordshire, UK). Recordings of single‐unit activity were made using a Neurolog extracellular recording system (NL104A, Digitimer, Hertfordshire, UK) and Spike 2 software (Cambridge Electronic Design, Cambridge, England). Extracellular spikes were digitized at 30 kHz after being amplified (1000×) and band‐pass filtered (600–6000 Hz) using a dedicated AC‐differential amplifier (NL104A, Digitimer, Hertfordshire, UK). The neuronal signals were also fed into a Grass Audioamplifier (AM8, Indianapolis, IN) for audio‐feedback during recording.

Electrodes were placed in an electrode holder and moved using a scientific micromanipulator (IVM‐3000, Scientifica, UK) under computer control (LinLab software, Scientifica, UK). The electrode was positioned on the surface of the brain using an otolaryngological microscope and then advanced through the striatum at a rate of between 1 and 2 *μ*m per second. The search was concentrated on, but not limited to, the dorsomedial striatum, both ipsilateral and contralateral to the stimulation. There were 3–4 recorded tracks made per animal, which were at least 300 *μ*m apart to allow searching in a variety of areas and also to ensure that the tracks did not affect one another. When neuronal spikes were seen in the trace, they were classified as either responsive or nonresponsive to the vestibular stimulation. If a neuron did not obviously respond to vestibular stimulation, that is, with a 3–4 spike/s change in spontaneous activity within several seconds, the electrode was advanced further.

#### Data Analysis

All data analysis was performed offline using Spike 2. All neurons that fired during recording were analyzed for wave classification and firing pattern. Cells were classified as either “M” units or “F” units as described by Berke et al. (2004). In brief, peak width and valley width were measured at half maximum. “M” units have firing rates of less than 5 Hz and a valley width of more than 300 *μ*s, whereas “F” units have a firing rate of greater than 2 Hz and a valley width of less than 265 *μ*s and a peak width of less than 120 *μ*s. “M” units were identified as likely to be medium spiny neurons whereas “F” units were identified as fast spiking neurons and were therefore likely to be interneurons (Berke et al. 2004). The firing patterns of the neurons were analyzed to determine the different types of responses seen. The neurons were divided into 5 categories: singles spikes, paired spikes, spike trains, single bursts, and burst trains (see Table 1).

**Table 1:** Classification of neuronal firing for neurons recorded in the striatum

Label	Definition
Singles spikes	A single spike with no other associated firing
Paired spikes	A single burst of firing with 2 spikes
Spike trains	Single spikes of the same neuron separated by a period of greater than 0.5 sec
Burst	A single burst with 3 or more spikes
Burst train	Multiple spike burst firing of the same neuron separated by a period of greater than 0.5 sec

Neurons were classified as responsive to vestibular stimulation if their firing was consistently phase‐locked to the vestibular stimulation. Responsive neurons were classified by cell type (either “M” or “F” units) and the latency of the response following the stimulation was measured from the last part of the stimulus artifact to the first spike of the responsive cells.

#### Histology for electrode placement

At the completion of the recording experiments, the whole brain was rapidly removed and placed in 10% formalin solution. Forty‐eight hours before sectioning, the brains were transferred to a phosphate buffer with 30% sucrose solution. The brains were cut into 40 *μ*m sections using a freezing microtome between −18°C and −20°C. Sections were incubated with 0.0015% cresyl violet diluted with 1 M acetic acid for 30 min. The slides were examined under a light microscope (Nikon Elipse, Ni‐E) to determine the electrode placement.

### c‐Fos study

#### Stimulation

For the c‐Fos study, a square wave stimulation train at 100 Hz (usually 300–400 *μ*A) for 10 min was used; in separate groups of animals from the elecrophysiological study, stimulation was delivered at 1×, or 2×, the nystagmus threshold. For sham control animals the surgical procedure was identical; however, the animals did not receive any vestibular stimulation and they were not tested for nystagmus.

#### Tissue collection and sectioning

Ninety minutes after the end of the stimulation, while still anesthetized, animals were euthanized and underwent tissue fixation via perfusion with 4% paraformaldehyde (PFA). The brains were dissected out, post‐fixed and frozen for sectioning. Forty‐micrometer coronal sections were collected throughout the basal ganglia using a systematic random collection method in order to allow for stereological counting (see below).

#### Immunohistochemistry

All steps were carried out at room temperature unless otherwise stated. Antigen retrieval was then performed by incubating the sections in citrate buffer (pH 6) at 90°C for 10 min. The sections were incubated with 5% heat‐inactivated normal goat serum (NGS, Sigma) for 2 h before being incubated with polyclonal rabbit anti‐c‐Fos primary antibody (1:2000, sc‐52; Santa Cruz Biotechnology, Inc.) overnight at 4°C. The NGS as well as the antibodies were prepared in 0.01 M PBS containing 1% bovine serum albumin (BSA, Glibco) and 0.2% Triton X‐100. Following the primary antibody incubation the sections were washed with a high salt antibody washing buffer (0.01 M PBS containing 2% low‐fat milk powder (Pams), 1% NaCl (BDH) and 0.5% Triton X‐100) and were incubated with 0.5% H_2_O_2_ in 0.01 M PBS for 10 min. The sections were then incubated with an HRP‐conjugated goat anti‐rabbit secondary antibody (1:400; SC‐2004; Santa Cruz Biotechnology, Inc.) for 2 h. The antibody complex was visualized using diaminobenzidine (DAB).

#### Stereological counting

In order to estimate the total number of c‐Fos‐positive cells in the striatum, an optical fractionator method was used. Systematic random sampling is superior to independent random sampling due to a reduction in the sampling variance. In independent random sampling, the variance has been shown to be proportional to 1/*n* for *n* samples whereas systematic random sampling will produce a variance approximately proportional to 1/*n* ^2^ when *n* systematically random samples are taken (West et al. 1991). In order to systematically sample random sections for counting, a random starting section was selected using a random number generator (https://www.random.org/), then every 18th section was included in the sample set. In order to determine the accuracy of the estimates, the variation in the counts was calculated. The estimate was considered to be accurate if the coefficient of error (CE) within the animal was less than half that of the observed coefficient of variation between the animals (CV) (Gundersen and Jensen 1987).

Sections were visualized under an Olympus microscope and cell counting was performed using the optical fractionator protocol of the StereoInvestigator software (Version 10; MBF Bioscience). All cell counting was performed using the 100× objective lens. The sampling protocol was optimized after trialing a number of counting frames, sampling frames, and section sampling fractions, in order to provide a replicable count. The count was accepted when two repeated counts of the same brain using different sets of sample sections produced a total cell count within 10% of each other. It was found that between 150 and 200 individual cells must be counted to produce a reliable estimate of the total number of cells in the striatum (ΣQ^−^). Briefly, a sampling frame of 330 × 330 *μ*m was placed onto the sections in a random systematic manner and this resulted in ~400 stops per brain. At each sampling stop, a 20 × 20 *μ*m counting frame was placed within the sampling frame and the number of c‐Fos‐positive cells was counted within each counting frame in a 7 *μ*m depth (i.e., the thickness of the counting frame) with a 10% guard zone (i.e., the distance from the top of the slice). A c‐Fos‐positive cell was counted only if it met the counting criteria described by West (1993), that is, it was within the counting frame or touching the inclusion lines but not the exclusion lines of the counting frame. Only cells exhibiting clear nuclear labeling were counted.

The total number of c‐Fos‐positive cells (*N*) in the striatum was determined from the number of c‐Fos‐positive cells counted (*ΣQ* ^*−*^), the section sampling fraction (*ssf* = 1/18th, every 18th section was counted), the thickness sampling fraction (*tsf*, the ratio between the thickness of the counting frame and the thickness of the section) and the area sampling fraction (*asf*, the ratio between the area of the counting frame and the area of the sampling frame), using the calculation below (Gundersen et al. 1988).

$$N=\sum Q^{-}\times \frac{1}{\mathrm{ssf}}\times \frac{1}{\mathrm{tsf}}\times \frac{1}{\mathrm{asf}}$$

#### Statistical analyses

The aim of the single neuron recording experiments was to determine whether or not single striatal neurons responded to electrical stimulation of the peripheral vestibular system. Consequently, the statistical analyses consisted of using *χ* ^2^ analyses to investigate the frequency of neuronal types and response patterns, and a Bayesian Markov Chain Monte Carlo (MCMC) simulation analysis to estimate a credible interval for the number of vestibular‐responsive neurons in the striatum. The *χ* ^2^ analyses were *χ* ^2^ Goodness‐of‐Fit tests carried out in SPSS 24. The Bayesian MCMC simulations were performed using R (version 3.4.0), R Studio (version 1.0.143) as well as the R package, rjags (version 4.6), which allowed the use of JAGS within R (Lesaffre and Lawson 2012). Ten thousand simulations were used. The proportion of responsive cells was modeled as a binomial variable with *P *= probability and *n *= no. of trials, following a beta distribution. A Bayesian 95% credible interval was calculated. Since there were no comparable previous data, the prior values were set to (1,1) (Lesaffre and Lawson 2012).

For the c‐Fos study, data were tested to determine whether they fulfilled the normality assumption, natural log transformed if they did not, and then re‐tested. A linear mixed model (LMM) analysis was performed on the data using SPSS 24, with the treatment as a between‐group factor and the side as a repeated measure (McCulloch et al. 2008). Post hoc comparisons were conducted using Bonferroni‐adjusted t tests for multiple comparisons (McCulloch et al. 2008).

## Results

### Single neuron recording study

From 20 rats, 507 single neurons were recorded in total. Four hundred and fifty‐four neurons recorded were M units, and 53 were F units (*χ* ^2^ (1) = 317.46, *n *=* *507, *P *≤* *0.0001). There were significant differences in the response types (*χ* ^2^ (4) = 138.37, *n *=* *513, *P *≤* *0.0001).

**Figure 3:**
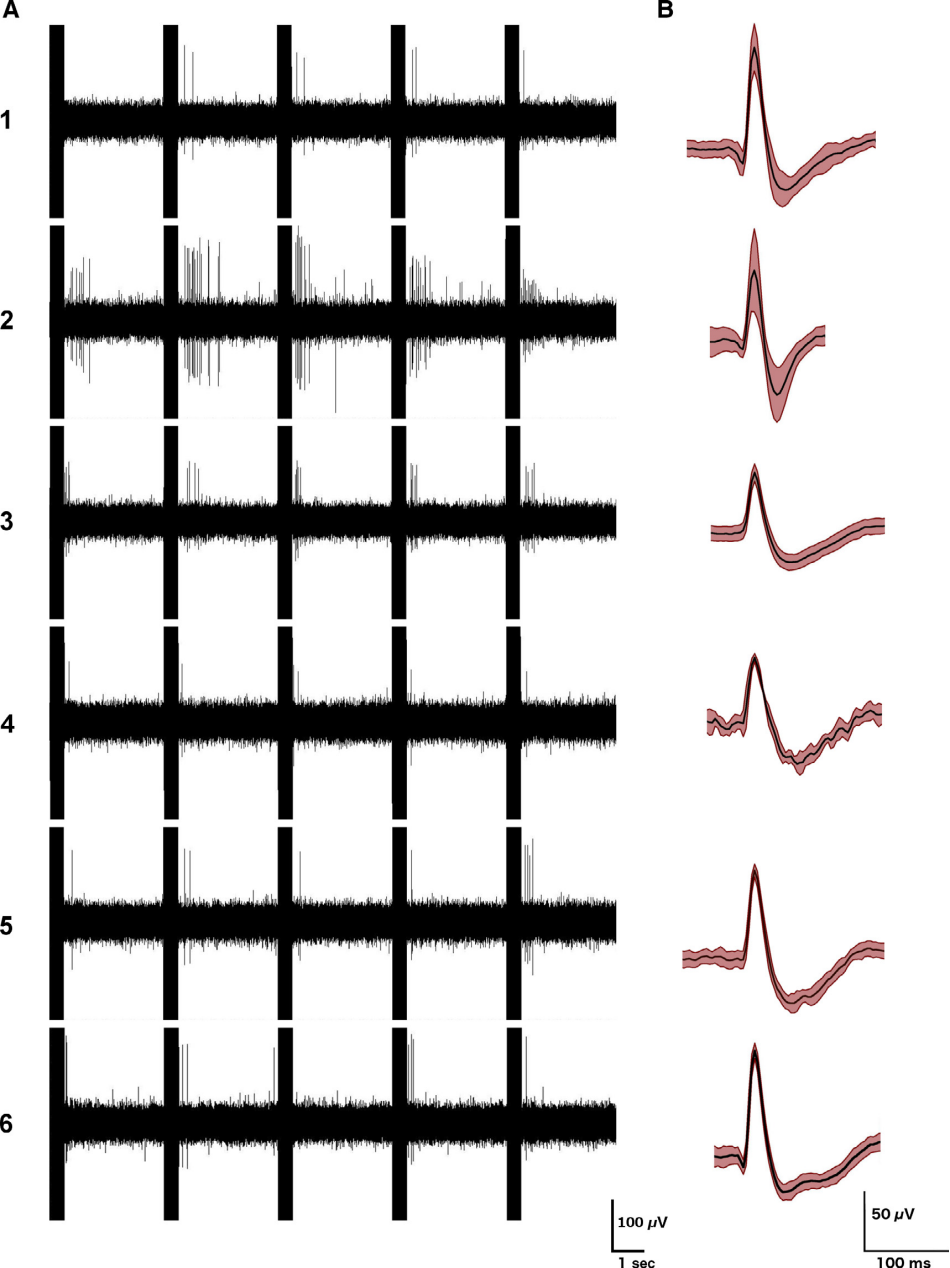
Examples of the firing patterns of the 6 single striatal neurons responding to electrical stimulation of the vestibular labyrinth in a phase‐locked manner, with examples of their action potential waveforms (averages of 200 action potentials; mean ± SD in red).

**Figure 4:**
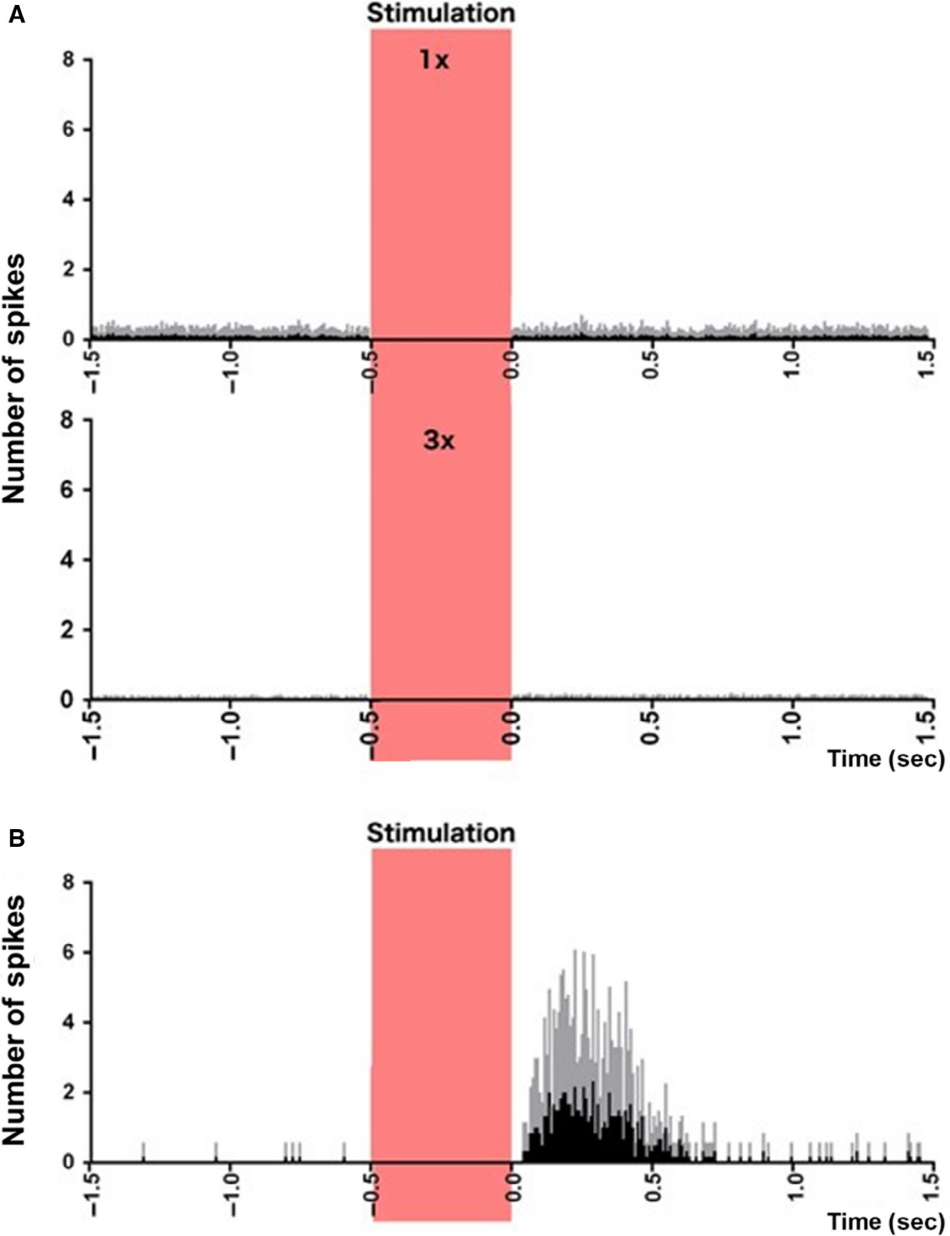
Peri‐stimulus histograms of neuronal responses to electrical vestibular stimulation. (A) Combined histogram of firing of all nonresponsive neurons at 1× (top) and 2× (bottom) the threshold of nystagmus. (B) Combined firing of all 6 responsive neurons, at 3× the threshold of nystagmus, phase‐locked to the stimulus. Red bar represents the stimulation period. Spikes from the stimulus artifact have been removed for clarity. Data are presented as mean (black bars) and standard deviation (gray bars).

No vestibular‐responsive neurons were found at the lower current amplitudes and only 6 were found in total at the highest current amplitude (6/507 or 1.1%). All of these neurons responded with an increase in firing rate; no obvious decreases were observed (Figs. 3 and 4). Three of these neurons were found on the ipsilateral side and 3 on the contralateral side. In these cases the firing rates of the neurons were tightly phase‐locked to the stimulus. Figure 3 shows the response of the 6 neurons as well as the waveforms of their action potentials. Four of these neurons were located 3.8 mm deep into the striatum and had the firing characteristics of medium spiny neurons. The average response latency of 5/6 was 50 msec from the end of the stimulus artifact (range: 33–84 msec) and the remaining neuron had a latency of 200 msec. Figure 4A shows a peri‐stimulus histogram of the mean combined firing of all nonresponsive neurons at 1**×** (top) and 3× (bottom) the threshold for nystagmus. Figure 4B shows the mean combined firing of all 6 responsive neurons at 3× the threshold for nystagmus, phase‐locked to the stimulus.

**Figure 5:**
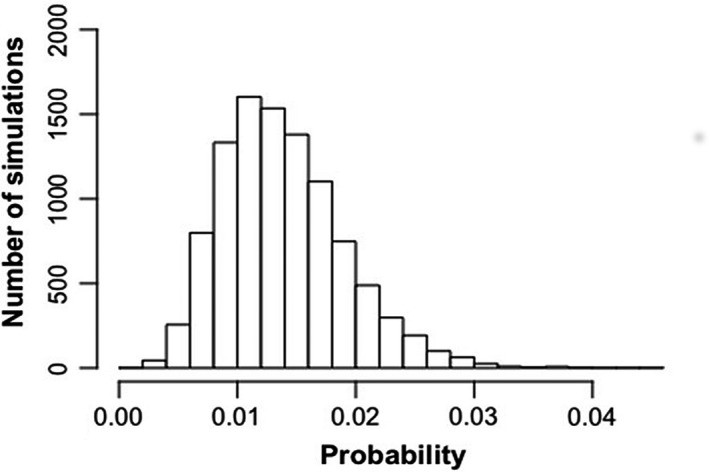
Histogram showing the results of 10,000 Bayesian MCMC simulations to estimate the frequency of vestibular‐responsive neurons in the striatum. The Bayesian credible interval was between 0.4% and 2.0%.

**Figure 6:**
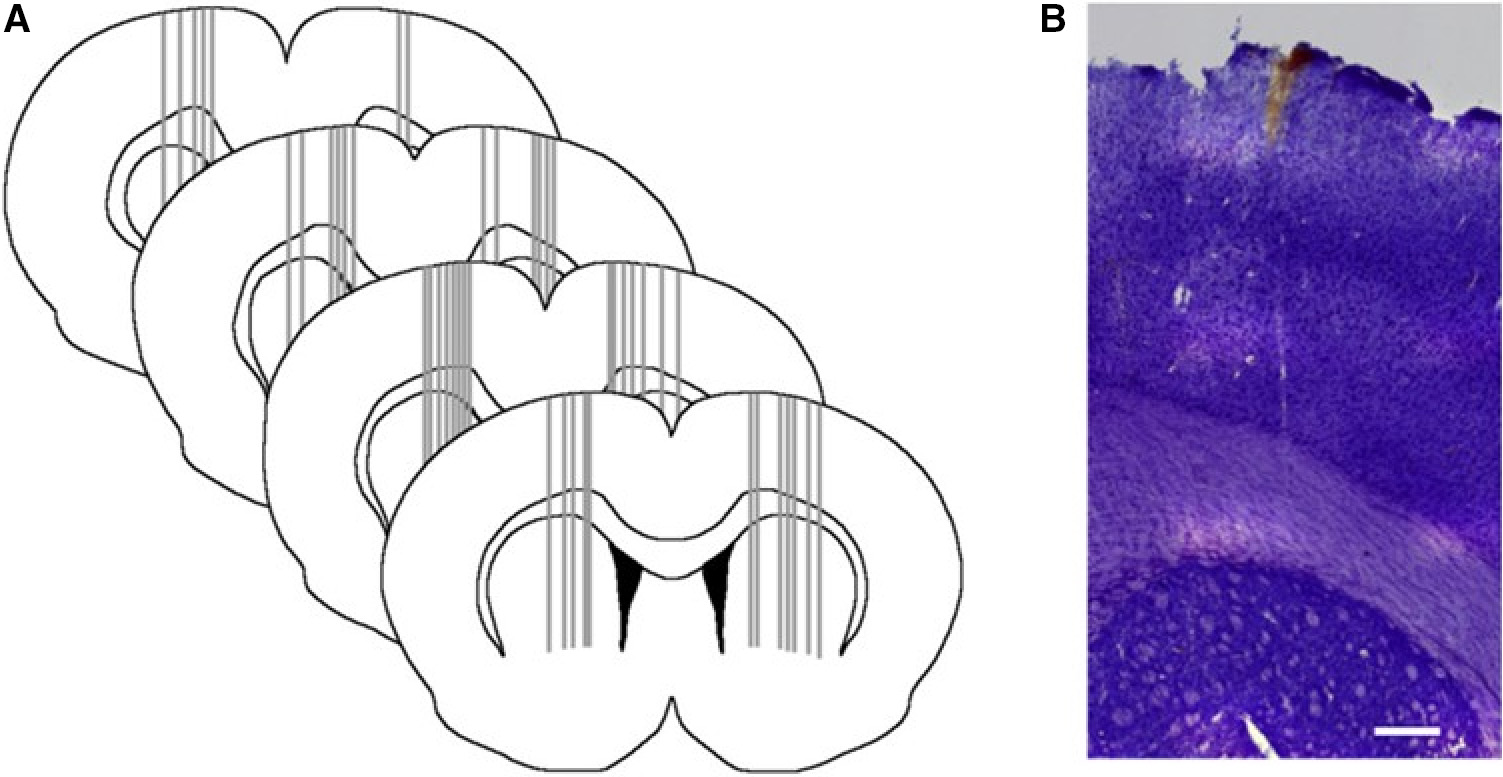
Schematic diagram showing the recording sites for the striatal neurons analyzed in the current study and an example of a cresyl violet‐stained section showing a typical electrode track. Scale bar = 20 *μ*m.

Based on 10,000 MCMC simulations, a Bayesian credible interval for the number of neurons responding to vestibular stimulation, was estimated to be between 0.004 and 0.02, indicating that the percentage of neurons responding to vestibular stimulation was between 0.4 and 2.0% (Fig. 5). Figure 6 shows the locations of the recording electrode sites.

### c‐Fos study

**Figure 7:**
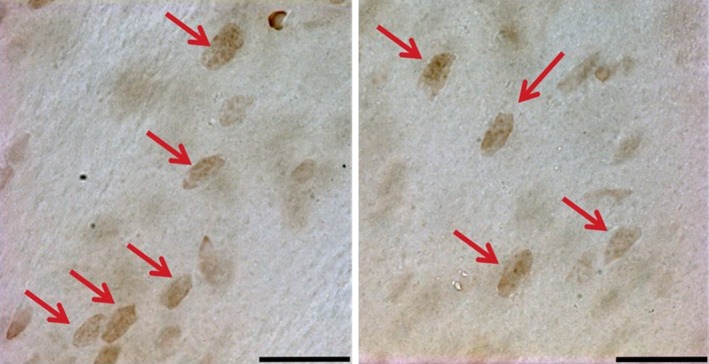
Example of c‐Fos labeling in the striatum. In order to be counted, cells had to have clear nuclear labeling. Scale bar = 20 *μ*m.

Positve c‐Fos labeling of cells in the striatum appeared as dark brown staining of the nucleus when examined under the microscope (see Fig. 7 for an example).

**Figure 8:**
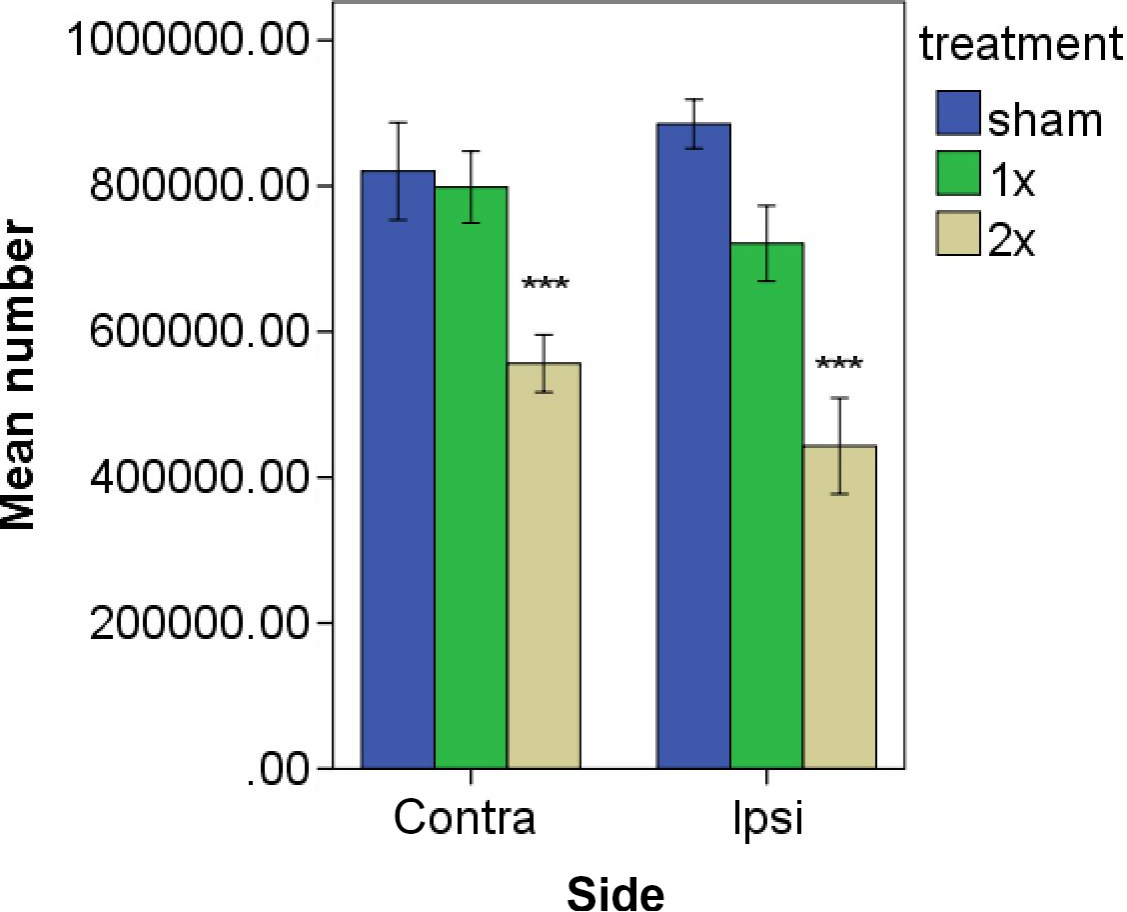
Estimated number of c‐Fos‐positive cells in the striatum following vestibular stimulation. ***P ≤* *0.0001 for the comparison of the higher current with both the sham groups and the lower current group *P *≤ 0.0001) (see Figs 4 and 5).

The LMM analysis demonstrated that the treatment resulted in a significant difference in the number of c‐Fos‐positive cells in the striatum (F(2,24) = 24.21, *P *≤* *0.0001; Fig. 8). However, neither the side (F(1,24) = 0.96, *P *=* *0.337), *P *≤* *0.38) nor the side x treatment interaction (F(2, 24) = 1.61, *P *=* *0.222), *P *≤* *0.22) was significant. Post hoc tests showed that the significant treatment effect was due to a reduction in the number of c‐Fos‐positive cells for the 2× stimulus intensity condition compared to both the sham control group (*P *≤* *0.0001) *P *≤* *0.0001) and the lower stimulus intensity condition (*P *≤* *0.0001; Fig. 8) *P *≤* *0.0001) (see Figs 4 and 5).

## Discussion

The results of this study have demonstrated that electrical stimulation of the vestibular labyrinth can evoke responses in single striatal neurons, albeit in a very small and circumscribed population. They have also demonstrated, for the first time, that such electrical stimulation can affect the number of cells expressing c‐Fos in the rat striatum. These results support the hypothesis that the vestibular system and the striatum are connected synaptically and that vestibular stimulation is likely to have an influence on neuronal function in the striatum (see Stiles and Smith 2015 for a review).

It must be considered whether the electrical stimulation employed would have caused other nonspecific effects through current spread. The use of a bipolar electrode should have minimized current spread. The stimulation intensities used were carefully calibrated to evoke vestibular nystagmus and this was confirmed using video‐microscopy (see Fig. 2). The lower intensity was the minimal necessary to induce nystagmus and the higher intensities, 3× that in the case of the electrophysiological study, and 2× that in the case of the c‐Fos study. It is conceivable that the changes in neuronal activity and c‐Fos expression in the striatum were mediated partly by effects on the auditory system. However, Rancz et al. (2015) used comparable stimulation of the rat superior vestibular nerve and found little evidence of auditory activation. Electrical stimulation of the round window has been reported not to activate single neurons in the auditory cortex or to induce auditory sensations in humans (e.g., Korhuber and DaFonseca 1964; Schwartzkroin 1973). Nonetheless, this possibility cannot be excluded in the present study. However, there is no question that the peripheral vestibular system was activated because this was demonstrated by the confirmation of vestibular nystagmus using video‐microscopy (see Fig. 2). It is unlikely that the striatal effects were due to the higher stimulus current causing sensory feedback from muscle contraction, because there was no evidence that the current intensities were high enough for this and whether movement occurred was carefully monitored.

Under anesthesia, the spontaneous firing of neurons in the striatum decreases significantly (Berke et al. 2004). From 507 neurons recorded in the electrophysiological study, only 6 neurons responded to electrical stimulation of the vestibular labyrinth and all of these responded only to the higher stimulus current, that is, 3× the threshold for nystagmus. These neurons, which appeared to be medium spiny neurons, were located equally on the sides ipsilateral and contralateral to the stimulation and the latencies were usually between 33–84 msec. Using Bayesian MCMC simulations, we estimated that the true frequency of vestibular‐responsive neurons in the striatum under urethane anesthesia was between 0.4% and 2% of the population, although our recordings were focused on the dorsomedial area of the striatum. To the best of our knowledge, there have been only two previous single neuron studies of striatal responses to electrical stimulation of the vestibular system, which leads to the impression that field responses are easier to obtain than single‐unit responses (Stiles and Smith 2015). Segundo and Machne (1956) reported that electrical stimulation of the vestibular labyrinth in cats caused an increase or a decrease in the firing rate of single neurons in the putamen and the globus pallidus in cats. The results were presented qualitatively, with no statistical analyses; however, the paper suggests that responses were infrequent. The current intensities employed were similar to the current study. On the other hand, Matsunami and Cohen (1975) found that electrical stimulation of the contralateral vestibular nucleus in awake rhesus monkeys did not cause any change in the firing rate of single striatal neurons in the caudate nucleus, except when stimulation trains were used and the current intensity was high enough to produce movement of the limbs. Again, the results were presented qualitatively with no statistical analyses and it was notable that although they found no single unit responses, they did still record field potential responses. In this study, we could find no evidence of decreased firing in response to vestibular stimulation, even when all of the neurons recorded were combined (Fig. 4A). In the previous studies, no quantitative comparison of the response of the different types of striatal neurons to vestibular stimulation was provided; therefore, it is impossible to compare the present results with those studies.

It is reasonable to ask that if the frequency of vestibular‐responsive neurons in the striatum, at least under urethane anesthesia, is between 0.4% and 2% of the neuronal population, do these responses really matter? It is impossible to answer this question based on the current data. However, it is worth noting that the cholinergic neurons of the striatum constitute only 1–3% of the total population and yet are critical to striatal function (Benarroch 2012). Whether vestibular responses are important to striatal function remains to be seen. However, indirect evidence from studies of the effects of vestibular activation and inactivation on locomotor hyperactivity (Stiles et al. 2012; Antoine et al. 2013; see Stiles and Smith 2015 for a review) and Parkinson's Disease (Yamamoto et al. 2005; Pan et al. 2008; Samoudi et al. 2012; Iwasaki et al. 2014), suggests that this is likely to be the case.

A surprising result from the c‐Fos study was that effects were obtained at the higher stimulus intensity and this resulted in a significant *decrease* in the number of cells expressing c‐Fos. c‐Fos has been reported to be expressed under basal conditions in the striatum (see Hughes and Dragunow 1995 for a review), and although anesthetics such as urethane have been reported to suppress the increase in striatal c‐Fos expression caused by cocaine, it had no effect on basal c‐Fos expression (Kreuter et al. 2004). Therefore, it is unlikely that the basal c‐Fos expression observed in this study was due to the effects of urethane. We are confident that the reduction in the number of cells expressing c‐Fos, as a result of vestibular stimulation, is a reliable result, because we employed stereological cell counting, which has been demonstrated to result in minimal bias in estimates of cell number (Gundersen and Jensen 1987; West et al. 1991). The 10 min duration of stimulation used for the c‐Fos study was initially based on previous studies of the effects of galvanic vestibular stimulation on cell proliferation in the hippocampus (Zheng et al. 2014), as well as pilot data. It was assumed that briefer stimulation may not be sufficient to alter c‐Fos expression in the striatum. The rats were sacrificed at 90 min poststimulation in order to provide sufficient time for the c‐Fos gene to be activated and the protein to be produced (Sheng and Greenberg 1990; Jaworski et al. 1999; see Kawashima et al. 2014 for a review). It is entirely possible that the results obtained are specific to these stimulus conditions and the time point chosen. Nonetheless, the decrease in the number of cells expressing c‐Fos was surprising and, ostensibly, suggests that increasing activation of the peripheral vestibular system results in reduced activation of the striatum. What might explain such an effect? One obvious possibility is that at higher stimulus intensities, activation of the vestibular nerve results in the recruitment of an inhibitory pathway, either via the vestibular nucleus or the cerebellum (since it also receives direct input from the vestibular nerve), and that, via several synapses, this leads to an inhibitory effect in the striatum. Even within the brainstem vestibulo‐ocular reflex pathways, it is well established that excitation of the vestibular nerve or vestibular nucleus neurons can result in inhibitory effects due to the excitation of intercalated inhibitory interneurons (e.g., Hikosaka et al. 1980; Nakao et al. 1980, 1982; Curthoys et al. 1981, 1984). What pathway this may be is difficult to hypothesize without further information. However, there is evidence that some type II neurons in the medial vestibular nucleus, which are usually inhibitory, can be activated by ipsilateral inhibitory type I neurons; such inhibitory type I neurons could project outside the vestibular nucleus, in which case activation of the ipsilateral vestibular nerve would cause increased inhibition (Curthoys et al. 1987; Smith and Curthoys 1988a,b). Another possibility is that reduced c‐Fos expression is not necessarily indicative of decreased activation. c‐Fos has been used extensively as a marker of neuronal activation in the CNS and there is considerable evidence to indicate that elevated c‐Fos expression reflects increased neuronal excitation (e.g., Jaworski et al. 1999; see Kawashima et al. 2014 for a review). On the other hand, neuronal excitation is not always associated with increased c‐Fos expression (e.g., Ludwig et al. 1997). Inhibitory neurons can also express c‐Fos during increased activation (e.g., Staiger et al. 2002); therefore, the decreased number of c‐Fos‐positive cells probably reflects a decrease in the activation of medium spiny neurons, since they are GABAergic inhibitory neurons representing ~95% of striatal neurons.

It was of interest that there was no significant difference between the effects of peripheral vestibular stimulation on neuronal activity or c‐Fos expression in the left and right striatum. Some previous studies have suggested that the contralateral striatum may receive greater vestibular input from the vestibular labyrinth (e.g., Segundo and Machne 1956; Spiegel et al. 1965). However, responses have been found bilaterally (Spiegel et al. 1965; Potegal et al. 1971) and Rancz et al. (2015) could find no evidence of laterality in the striatal responses to vestibular nerve stimulation. Therefore, the lack of difference in the changes in neuronal activity and c‐Fos expression found in this study are consistent with previous electrophysiological evidence in terms of the bilateral effects of vestibular stimulation on the striatum.

While the electrophysiological study showed that electrical stimulation of the peripheral vestibular system could evoked increases in firing in a small population of striatal neurons, the c‐Fos study suggested that electrical stimulation of the vestibular system reduced the number of cells in the striatum expressing c‐Fos, in a current‐dependent manner. How can these two sets of results be reconciled? While both sets of results support the hypothesis that the vestibular system and striatum are connected, it is difficult to relate them to one another at a detailed level. The electrophysiological study used brief stimulation at 3× the threshold whereas the c‐Fos study used current amplitudes at 1× and 2× the threshold for vestibular nystagmus for 10 min. The electrophysiological study involved recording sessions lasting many hours searching for neurons that would respond to stimulation in a phase‐locked fashion, while the c‐Fos study used one time point following stimulation. Whereas the c‐Fos study used stereological methods and therefore was subject to minimal sampling bias, electrophysiological recording of single neurons is inevitably biased by electrode impedance, the depth of anesthesia, the physiological state of the animal and whether responses have been found in particular areas. From this viewpoint it is possible that these two sets of results represent different populations of striatal neurons, although given the common occurrence of medium spiny neurons and the waveforms of the action potentials of neurons recorded, it is likely that both sets of results reflect that neuronal category. Nonetheless, taken at face value, the electrophysiological results suggest that activation at 3× the threshold causes increased activity, albeit in a small group of neurons, whereas the c‐Fos results suggest that activation of the vestibular system at 2× the nystagmus threshold causes reduced striatal activity. One possibility is that many neurons exhibited a decrease in firing following electrical stimulation of the vestibular labyrinth but that, under urethane anesthesia, this could not be detected because the spontaneous firing rate was so low that decreases could not be detected. Segundo and Machne (1956) did report that some neurons exhibited a decrease in firing rate in response to vestibular stimulation; however, this was not quantified. It is possible that the few neurons that showed an increase in firing were the minority and that the main effect of activation of the vestibular system was to *decrease* striatal activity. Although vestibular stimulation has been reported to cause activation of the basal ganglia in humans (Bottini et al. 1994; Vitte et al. 1996; Emri et al. 2003; Della‐Justina et al. 2014), Jahn et al. (2004) found that this activation was reduced during imagined walking compared to imagined standing and that imagined running was not associated with basal ganglia activation. Recordings from striatal neurons in alert behaving animals suggest that neuronal activity is likely to be very specific to particular activities during locomotion in a behavioural task (Barnes et al. 2005). Reduced neuronal activation during electrical stimulation of the vestibular labyrinth might partially explain why field potential responses have generally been easier to record than responses from single neurons (Spiegel et al. 1965; Potegal et al. 1971; Matsunami and Cohen 1975; Liedgren and Schwarz 1976; Rancz et al. 2015). However, we could find no evidence of a decrease in firing, either at the level of a single neuron or by averaging across the sample of single neurons recorded.

It is difficult to speculate about the likely route of transmission of vestibular information to the striatum from the activation of the vestibular labyrinth. Most of the responses found had an average latency of 50 msec. Schulz et al. (2009) have reported that, under urethane anesthesia, striatal responses to visual stimuli, transmitted through 3 synapses, had a latency of ~150 msec. Therefore, the latencies found in this study could be consistent with direct disynaptic pathways from the vestibular nucleus to the striatum, for example, via the PFN (Lai et al. 2000). However, since the cerebellum receives direct input from the vestibular nerve, it is possible that some of the vestibular input arises from the cerebellum rather than the vestibular nucleus, for example, via the pedunculopontine tegmental nucleus (PPT), whose neurons are vestibular‐responsive (Aravamuthan and Angelaki 2012) and which project to the striatum (Kobayashi and Nakamura 2003).
